# Up-regulation of the kinase gene *SGK1* by progesterone activates the AP-1–NDRG1 axis in both PR-positive and -negative breast cancer cells

**DOI:** 10.1074/jbc.RA118.002894

**Published:** 2018-10-18

**Authors:** Mukul Godbole, Trupti Togar, Kuldeep Patel, Bhasker Dharavath, Neelima Yadav, Sharan Janjuha, Nilesh Gardi, Kanishka Tiwary, Prachi Terwadkar, Sanket Desai, Ratnam Prasad, Hemant Dhamne, Kunal Karve, Sameer Salunkhe, Dhananjay Kawle, Pratik Chandrani, Shilpee Dutt, Sudeep Gupta, Rajendra A. Badwe, Amit Dutt

**Affiliations:** From the ‡ Integrated Cancer Genomics Laboratory and; the ¶ Shilpee Laboratory, Advanced Centre for Treatment, Research, and Education in Cancer,; the ‖ Department of Medical Oncology, and; the ** Department of Surgical Oncology, Tata Memorial Hospital, Tata Memorial Centre, Navi Mumbai, Maharashtra 410210, India and; the § Homi Bhabha National Institute, Training School Complex, Anushakti Nagar, Mumbai, Maharashtra 400094, India

**Keywords:** progesterone, breast cancer, functional genomics, cell signaling, microRNA (miRNA), N-Myc-downstream regulated gene 1 (NDRG1), post-transcriptional regulation, Serum- and glucocorticoid-regulated kinase 1 (SGK1), transcription factor AP-1

## Abstract

Preoperative progesterone intervention has been shown to confer a survival benefit to breast cancer patients independently of their progesterone receptor (PR) status. This observation raises the question how progesterone affects the outcome of PR-negative cancer. Here, using microarray and RNA-Seq–based gene expression profiling and ChIP-Seq analyses of breast cancer cells, we observed that the serum- and glucocorticoid-regulated kinase gene (*SGK1*) and the tumor metastasis–suppressor gene N-Myc downstream regulated gene 1 (*NDRG1*) are up-regulated and that the microRNAs *miR-29a* and *miR-101-1* targeting the 3′-UTR of *SGK1* are down-regulated in response to progesterone. We further demonstrate a dual-phase transcriptional and post-transcriptional regulation of *SGK1* in response to progesterone, leading to an up-regulation of *NDRG1* that is mediated by a set of genes regulated by the transcription factor AP-1. We found that *NDRG1*, in turn, inactivates a set of kinases, impeding the invasion and migration of breast cancer cells. In summary, we propose a model for the mode of action of progesterone in breast cancer. This model helps decipher the molecular basis of observations in a randomized clinical trial of the effect of progesterone on breast cancer and has therefore the potential to improve the prognosis of breast cancer patients receiving preoperative progesterone treatment.

## Introduction

The increasing complexity of multicellular organisms correlates with the increasing number of microRNAs rather than the number of coding genes encoded by the genome (1, 2), reflecting a gradual increase in the extent and intricacy of gene regulation (3). Hierarchically, microRNAs function downstream of transcriptional regulation of genes because microRNAs repress post-transcription of mRNAs (4). However, emerging evidence suggests that transcriptional and post-transcriptional regulation is often highly coordinated (5, 6). Hormones, for instance, have been hypothesized to regulate expression of target genes at the transcriptional and post-transcriptional level (7, 8). Estrogen up-regulates the expression of progesterone receptor (PR)^4^ by transcriptionally recruiting estrogen receptor (ER) at the promoter and, post-transcriptionally, by silencing expression of microRNAs targeting the 3′-UTR of *PR* in breast cancer cells (9). A similar example for the *ATP1B1* gene has been reported (10). However, systematic approaches to discern dual-regulated molecular targets of hormones in breast cancer remains poorly understood.

Understanding the molecular basis of clinical phenomena in response to therapeutic interventions has been an important point of intersection between medical and biological sciences. Whereas the clinical benefit of preoperative endocrine therapy is well documented in the literature (11, 12), more recently, we described the first randomized trial with preoperative progesterone resulting in greater than 10% absolute improvement in 5-year disease-free survival among node-positive breast cancer patients (13). Of several hypothesis-generating results from this study, the impact of progesterone on PR-negative patients particularly lends itself to a systematic characterization of molecular changes that progesterone may induce in breast cells.

Gene expression studies probing the targets of progesterone have been performed either restrictively in PR-positive breast cancer cell lines or in the presence of other hormones (14–18). Although few studies suggest a beneficial effect of progesterone, progesterone-responsive genes in PR-negative cells have not been studied (14, 15, 17, 19). To identify targets of progesterone independent of PR status of cells, we set out to perform an integrated genomic profiling of a panel of PR-positive and PR-negative breast cancer cell lines treated with progesterone, followed by functional analysis of the components found to be significantly altered. This study details the molecular action of progesterone on breast cancer cells, mediated by the up-regulation of a genomic axis inclusive of a tumor metastasis suppressor gene in breast cancer, independent of the PR status of cells.

## Results

### Gene expression analyses reveal a novel dual-phase regulation of SGK1 by progesterone in breast cancer cells

**Figure 1.**
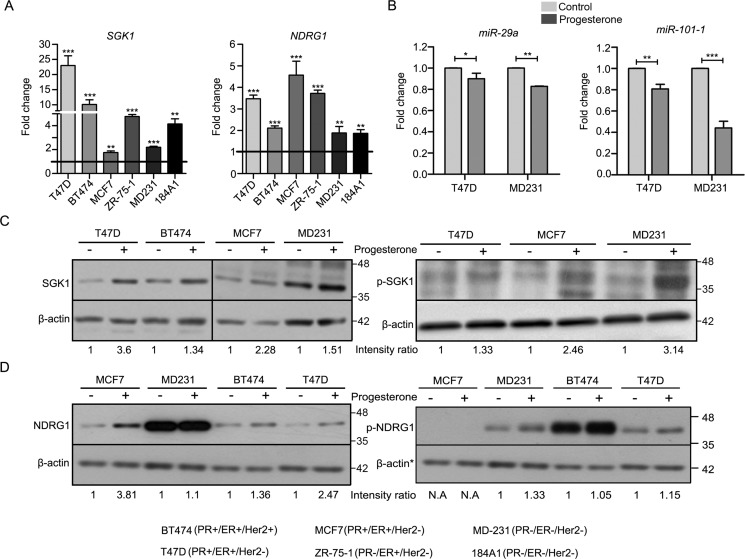
**Validation of expression of *SGK1* and *NDRG1*, and *miR-29a* and *miR-101-1* expression in breast cell lines treated with progesterone.** *A*, quantitative real-time PCR analysis for validation of expression of *SGK1* and *NDRG1* transcripts in breast cell lines in response to progesterone. Expression of both of the genes was normalized with respect to expression of *GAPDH* in each cell line. Data are plotted as -fold change for each gene with respect to the expression in control sample of each cell line. *Horizontal black line*, gene expression in control cells. The figure is representative of three independent experiments performed in triplicates. *p* value was calculated using Student's unpaired *t* test. *, *p* < 0.05; **, *p* < 0.005; ***, *p* < 0.0005. *B*, transcript levels of *miR-29a* and *miR-101-1* were measured using real-time PCR analysis in T47D and MDA-MB-231 cells treated with progesterone. The graph is plotted as expression -fold change of the two microRNAs normalized to expression of *U6* small RNA in progesterone-treated *versus* control cells. Transcript levels in both control and progesterone-treated cells are shown. The figure is representative of two independent experiments performed in triplicates. *C*, Western blot analysis for SGK1 (*left*) and p-SGK1 (*right*) in breast cancer cells treated with progesterone. *Minus sign*, control; *plus sign*, progesterone-treated samples. β-Actin was used as an internal loading control. *Numbers* on the blot indicate intensity ratio for SGK1 and p-SGK1, normalized to β-actin levels in the respective cell lines. The Western blot analyses for SGK1 and p-SGK1 are representative of three independent experiments. *D*, Western blot analysis of NDRG1 (*left*) and p-NDRG1 (*right*) in breast cancer cells treated with (+) and without (−) progesterone. *, β-actin protein was used as a loading control for Western blotting and is common for both of the blots, shown twice. *Numbers* on the blot indicate intensity ratio for NDRG1 normalized with respect to β-actin levels, whereas p-NDRG1 levels have been normalized with respect to total NDRG1 expression. The Western blot analysis is representative of three independent experiments. *Error bars* indicate S.D.

**Figure 2.**
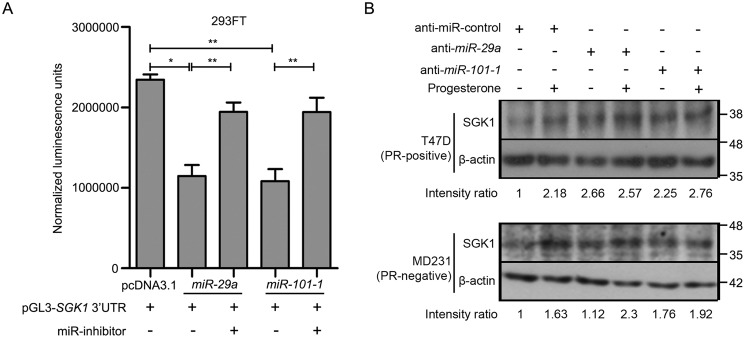
**Functional validation of *miR-29a*– and *miR-101-1*–mediated regulation of expression of *SGK1*.** *A*, quantification of luminescence units normalized to *Renilla* luciferase activity is plotted for pCDNA3.1-*miR-29a* or pCDNA3.1-*miR-101-1* and pGL3-*SGK1* 3′-UTR in different combinations with anti-*miR-29a* or anti-*miR-101-1* in 293FT cells. The figure is representative of three independent experiments performed in triplicates. *p* value was calculated using Student's unpaired *t* test. *, *p* < 0.05; **, *p* < 0.001; ***, *p* < 0.0001. *B*, Western blot analysis of SGK1 in T47D (PR-positive, *top*) and MDA-MB-231 (PR-negative, *bottom*) treated with anti-miR-control, anti-*miR-29a*, or anti-*miR-101-1*. As indicated in the *panel*, cells were either treated with progesterone or untreated. β-Actin was used as an internal protein-loading control. *Numbers* on the blot indicate intensity ratio of expression of SGK1 with respect to the *anti-miR-control lane* and expression normalized with respect to individual β-actin levels. The figure is representative of three independent experiments. *Error bars* indicate S.D.

An integrated analysis of microarray-based mRNA expression profile and deep sequencing of noncoding small RNA of breast cancer cells (as described under “Experimental procedures”) led us to identify up-regulation of a serum- and glucocorticoid-regulated kinase gene (*SGK1*) and N-Myc downstream regulated gene 1 (*NDRG1*), along with down-regulation of *miR-29a* and *miR-101-1*, predicted to bind the 3′-UTR of *SGK1*, independent of the hormonal receptor status of the cells (Fig. S1*A* and Tables S1 and S2). The up-regulation of *SGK1*, known to harbor multiple progesterone response elements (PREs) (20, 21), and *NDRG1* were observed to be relatively higher among the PR-positive cells, whereas *miR-29a* and *miR-101-1* were lower in PR-negative cells in response to progesterone (Fig. 1, *A–D*). Also, knockdown of *PR* significantly decreased the progesterone-induced up-regulation in expression of *SGK1* in PR-positive cells (Fig. S2*C*). Moreover, *SGK1* showed an increased expression based on analysis of the RNA-Seq data, reported earlier (15) (Fig. S2*B*), along with differentially enriched binding of PR and p300 at the *SGK1* loci in response to progesterone treatment, based on ChIP-Seq data (15) analysis (Fig. S2*A*), in T47D and MCF7 PR-positive cells (as explained under “Experimental procedures”). Interestingly, SGK1 activation (induction of p-SGK1 in response to progesterone) was found to be comparable in breast cancer cells, regardless of their PR status (Fig. 1*C*). Next, we validated *SGK1* as a target of *miR-29a* and *miR-101-1* by co-expressing the microRNAs along with firefly luciferase reporter genes cloned upstream to 3′-UTR of *SGK1*. Ectopic expression of both of the microRNAs decreased the firefly luciferase activity in 293FT cells expressing the 3′-UTR of *SGK1*. Consistent with the findings, transfection with anti-miRs targeting *miR-29a* and *miR-101-1* not only rescued the repression of luciferase activity in 293FT cells (Fig. 2*A*) but also led to sustained expression levels of SGK1 based on Western blot analysis of breast cancer cells (Fig. 2*B*). Taken together, the data suggest convergence of the dual mode of regulation at *SGK1* in response to progesterone treatment, along with up-regulation of *NDRG1* in multiple breast cancer cell lines independent of their PR status.

### SGK1 overexpression mimics progesterone treatment to up-regulate NDRG1

**Figure 3.**
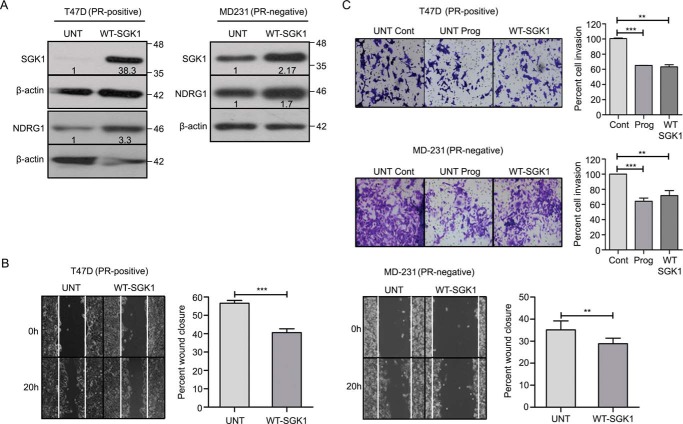
**Ectopic expression of *SGK1* mimics the effect of progesterone in breast cancer cells.** *A*, Western blot analysis indicating expression of SGK1 and NDRG1 in T47D (PR-positive, *left*) and MDA-MB-231 cells (PR-negative, *right*) overexpressing *SGK1*. β-Actin was used as an internal loading control. *Numbers* on the blot indicate intensity ratio for SGK1 and NDRG1, normalized to respective β-actin levels. The analysis is representative of three independent experiments. *B*, cell migration of T47D (*left*) and MDA-MB-231 (*right*) cells overexpressing *SGK1* was compared with untransfected parent cells in a wound scratch assay. The bar plots indicate percentage cellular migration of the cells, with a direct comparison between untransfected cells and cells overexpressing *SGK1*, and the analysis is representative of three independent experiments performed in triplicates. *C*, in cells overexpressing *SGK1*, cell invasion was studied in T47D (*top*) and MDA-MB-231 (*bottom*), and percentage cell invasion was compared with respective parent cells. Parent cells treated with progesterone were also used to compare the level of cell invasion upon *SGK1* overexpression. The bar plot depicts percentage cell invasion, and the figure is representative of three independent experiments performed in triplicates. *p* value was calculated using Student's unpaired *t* test. **, *p* < 0.005; ***, *p* < 0.0005. *Error bars* indicate S.D. *UNT*, untransfected.

**Figure 4.**
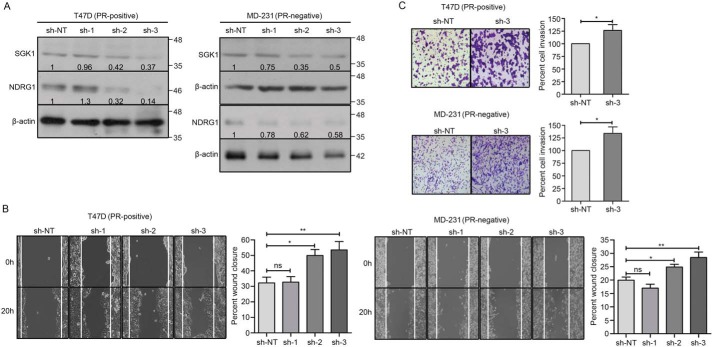
**Knockdown of *SGK1* decreases expression of *NDRG1* and increases cell migration and invasion in breast cancer cells.** *A*, Western blot analysis depicting expression of SGK1 and NDRG1 in T47D (PR-positive, *left*) and MDA-MB-231 (PR-negative, *right*) upon depleting the expression of *SGK1*. Expression of SGK1 and NDRG1 for each knockdown clone was compared with expression in non-targeting shRNA (*sh-NT*) clone. β-Actin was used as a loading control. *Numbers* on the blot indicate intensity ratio for expression of SGK1 and NDRG1, normalized to respective β-actin levels. The analysis is representative of three independent experiments. *B*, cell migration was studied upon knockdown of *SGK1* in T47D (*left*) and MDA-MB-231 (*right*) cells. The distance traversed by migrating cells was calculated from the start point to the migrated point over a period of 20 h. Data are plotted as percentage wound closure, and the figure panel is representative of three independent experiments performed in triplicates. *C*, invasion assay upon depletion of *SGK1* as compared with sh-NT clone of T47D (*top*) and MDA-MB-231 (*bottom*) cells, respectively. The bar plot represents percentage cell invasion with respect to invasion in sh-NT clone. The analysis is representative of three independent experiments performed in triplicates. *p* value was calculated using Student's unpaired *t* test. *, *p* < 0.05; **, *p* < 0.005; ***, *p* < 0.0005; *ns*, not significant. *Error bars* indicate S.D.

**Figure 5.**
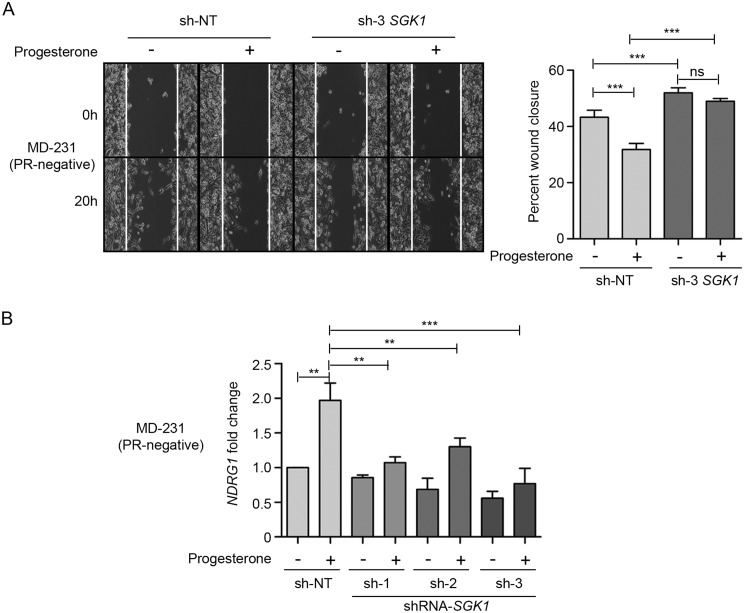
**Depletion of *SGK1* renders breast cancer cells partially responsive to progesterone.** *A*, cell migration assay upon depletion of *SGK1* in MDA-MB-231 cells (PR-negative), in the presence and absence of progesterone treatment. Cells were monitored by a time-lapse wound-healing assay for 20 h. Cell migration from the 0- to 20-h time point is plotted as percentage wound closure, and the comparison was with respect to sh-NT clone. The bar plot indicates percentage wound closure for each of the clones, treated with or without progesterone, and the quantification is an average of three independent experiments performed in triplicates. *B*, transcript levels of *NDRG1* have been analyzed in MDA-MB-231 cells (PR-negative) upon depletion of *SGK1*, in the presence and absence of progesterone stimulation. Data are plotted as -fold change of *NDRG1* with respect to expression in untreated sh-NT cells and individual *SGK1* knockdown clones. *GAPDH* was used as an internal normalization control. The analysis is representative of three independent experiments performed in triplicates. *p* value was calculated using Student's unpaired *t* test. **, *p* < 0.001; ***, *p* < 0.0001; *ns*, not significant. *Error bars* indicate S.D.

**Figure 6.**
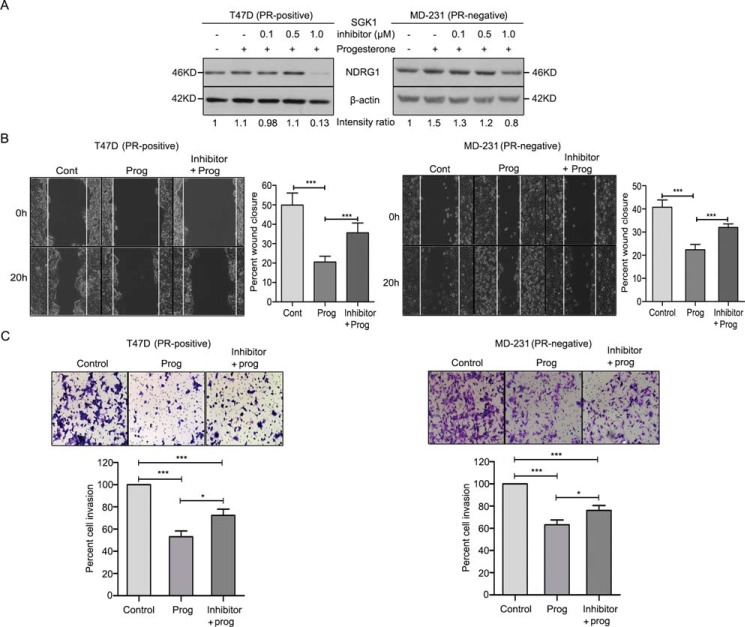
**SGK1 inhibitor phenocopies the effect of depletion of *SGK1* in breast cancer cells.** *A*, Western blot analysis representing the expression of NDRG1 in T47D (PR-positive) and MDA-MB-231 cells (PR-negative) treated with the SGK1 inhibitor and progesterone. Expression of NDRG1 was normalized with respect to β-actin levels in the respective cell lines. The *numbers* on the blot indicate the intensity ratio of expression of NDRG1 with respect to untreated cells in each cell line. The Western blot analysis is representative of three independent experiments. *B*, cell migration assay of breast cancer cells treated with *SGK1* inhibitor and progesterone. The motility of cells from the initial to the 20-h time point is plotted as percentage cell migration, and the comparison was between control, progesterone-treated, and SGK1 inhibitor + progesterone–treated conditions. The bar plot indicates percentage wound closure in each of the three treatment conditions. Analysis is representative of three independent experiments performed in triplicates. *C*, cellular invasion assay was performed with *SGK1* inhibitor treatment in T47D and MDA-MB-231 cells. *Panels* show cells with no treatment, cells with progesterone treatment, and cells with both inhibitor and progesterone combination treatment. The bar plot represents percentage cell invasion for each of the treatment conditions. The figure is representative of three independent experiments performed in triplicates. *p* value was calculated using Student's unpaired *t* test. *, *p* < 0.05; ***, *p* < 0.0005. *Error bars* indicate S.D.

*SGK1*, when overexpressed in PR-positive T47D and PR-negative MDA-MB-231 breast cancer cells, mimicked the effect of progesterone by up-regulating the expression of *NDRG1* (Fig. 3*A*), which in turn led to a significant reduction in cell migration and cell invasion (Fig. 3, *B* and *C*). In a reciprocal approach, depletion of *SGK1* in T47D and MDA-MB-231 cells led to a decrease in expression of *NDRG1* (Fig. 4*A*) with an inverse effect observed on migration and invasion of the breast cancer cells (Fig. 4, *B* and *C*), regardless of progesterone treatment (Fig. 5, *A* and *B*). Furthermore, consistent with the genetic perturbation, pharmacological inhibition of SGK1 with 1 μm GSK650394A similarly blocked the effect of progesterone on breast cancer cell migration and cell invasion, suggesting an essential role of the *SGK1*/*NDRG1* axis downstream to progesterone in breast cancer cells independent of their hormonal receptor status (Fig. 6).

### AP-1 transcription factors mediate up-regulation of NDRG1

**Figure 7.**
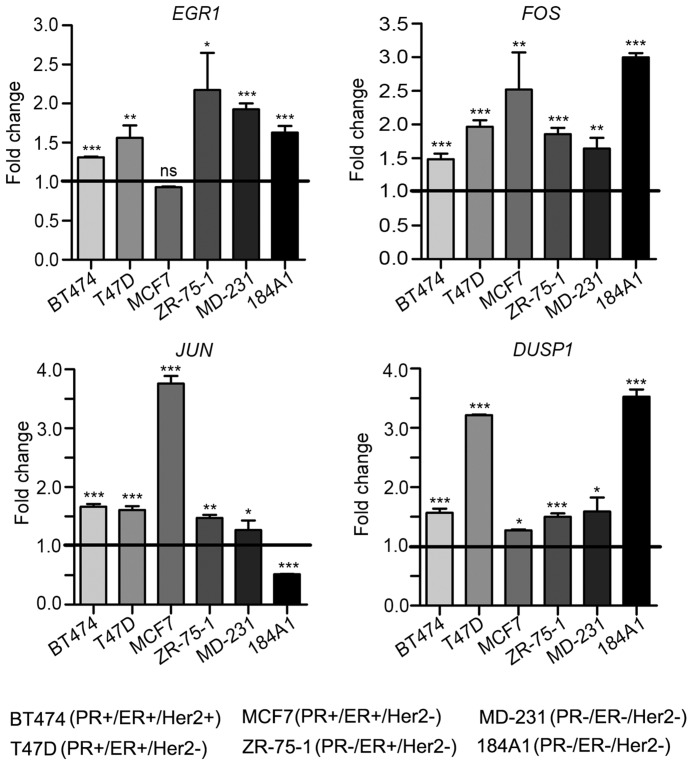
**Progesterone up-regulates expression of the AP-1 network genes in breast cell lines.** In the panel of breast cell lines representing different receptor status, transcript levels of AP-1 network genes (*EGR1*, *FOS*, *JUN*, and *DUSP1*) were studied using quantitative real-time PCR analysis. *Horizontal black line*, expression of individual genes in the control sample of respective cell line. Data are plotted as -fold change with respect to expression in control after normalization to expression of *GAPDH*. The figure is representative of three independent experiments performed in triplicates. *p* value was calculated using Student's unpaired *t* test. *, *p* < 0.05; **, *p* < 0.001; ***, *p* < 0.0001; *ns*, not significant. *Error bars* indicate S.D.

**Figure 8.**
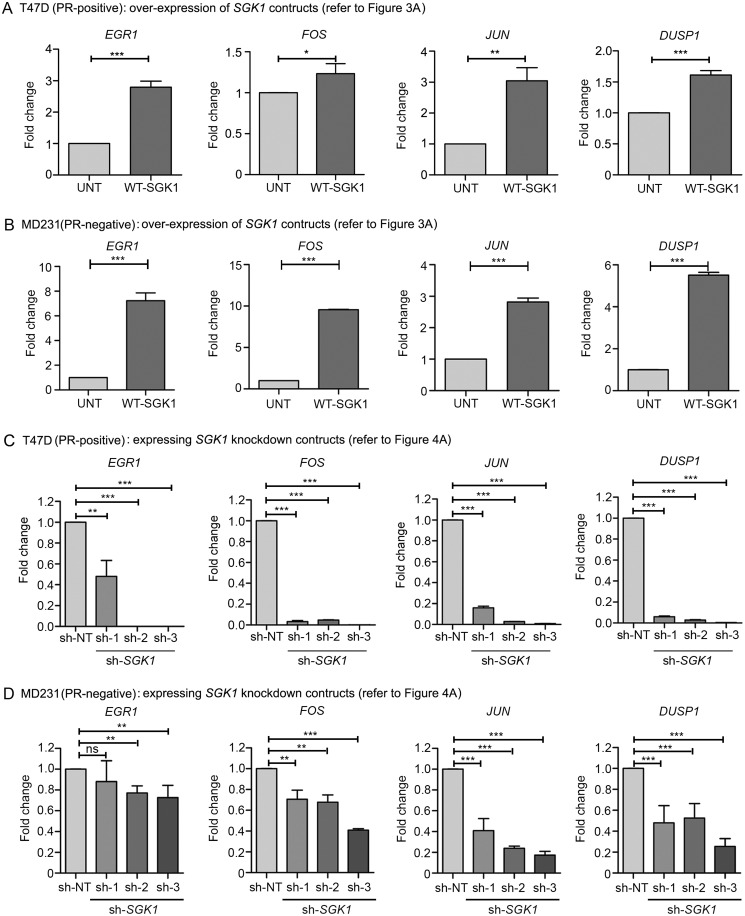
**SGK1 regulates expression of the AP-1 network genes in breast cancer cells.** Transcript levels of AP-1 network genes (*EGR1*, *FOS*, *JUN*, and *DUSP1*) were studied using quantitative real-time PCR in T47D (PR-positive) and MD231 (PR-negative) overexpressing *SGK1* (*A* and *B*) and upon knockdown of *SGK1* (*C* and *D*) in both of the cell lines, respectively. Gene expression analysis upon overexpression of *SGK1* is plotted compared with the untransfected cells. In the case of analysis upon knockdown of *SGK1*, transcript levels of AP-1 network genes were compared against sh-NT clone. Data are plotted as -fold change for each individual gene with respect to expression in sh-NT clone and normalized with respect to *GAPDH*. Both of the real-time PCR analyses are representative of three independent experiments performed in triplicates. *p* value was calculated using Student's unpaired *t* test. *, *p* < 0.05; **, *p* < 0.005; ***, *p* < 0.0005. *Error bars* indicate S.D.

**Figure 9.**
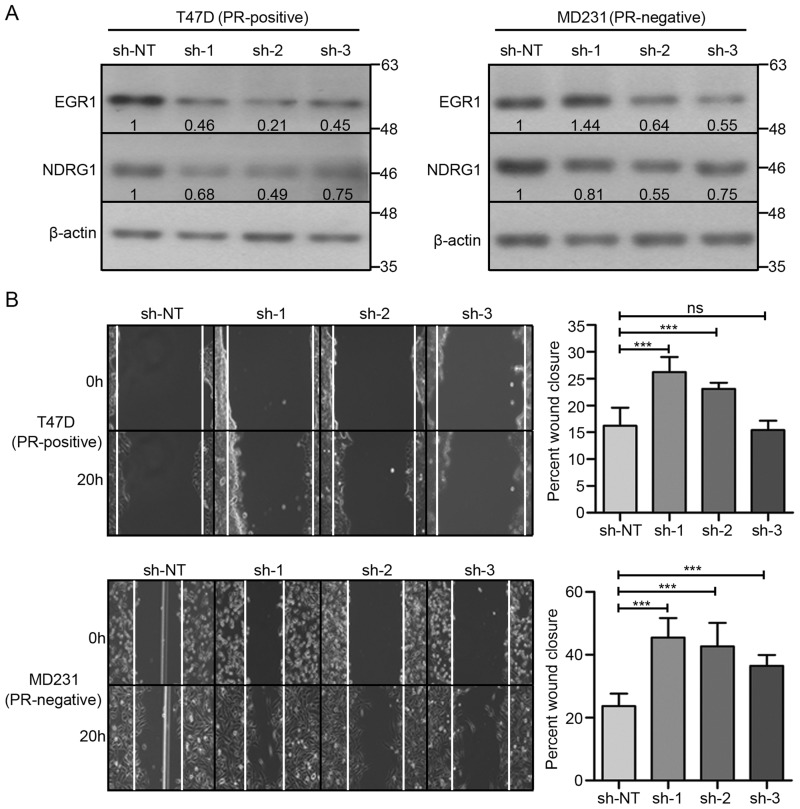
**Knockdown of *EGR1* decreases expression of *NDRG1* in breast cancer cells.** *A*, Western blot analysis of EGR1 and NDRG1 in T47D (PR-positive, *left*) and MDA-MB-231 (PR-negative, *right*) cells upon genetic depletion of *EGR1*. sh-NT was used as vector control. β-Actin protein was used as a loading control for Western blotting. *Numbers* on the blot indicate the intensity ratio for expression of EGR1 and NDRG1, normalized to respective β-actin levels. Western blot analysis is representative of three independent experiments. *B*, cell migration analysis upon depletion of *EGR1* in T47D (*top*) and MDA-MB-231 (*bottom*) breast cancer cells. Cells were monitored by a time-lapse wound-healing assay for 20 h. Cell migration from the 0- to 20-h time point is plotted as percentage wound closure, and the comparison was with respect to the sh-NT clone. The analysis is representative of three independent experiments performed in triplicates. *p* value was calculated using Student's unpaired *t* test. ***, *p* < 0.0001; *ns*, not significant. *Error bars* indicate S.D.

*NDRG1* is known to be regulated by AP-1 (*FOS*/*JUN*) and *EGR1* in response to stress-induced activation of kinases such as *p38*, *JNK*, and *ERK* (22–25). We recently showed that progesterone modulates the effect of surgical stress in primary breast cancer patients (26). Thus, we asked whether *NDRG1* could be regulated by AP-1 network genes in response to progesterone-induced activation of *SGK1* in a similar manner in breast cancer cells. Indeed, treatment with progesterone or overexpression of *SGK1* led to severalfold overexpression of the AP-1 network genes in a panel of breast cell lines irrespective of their PR status (Figs. 7 and 8 (*A* and *B*)). Consistent with this finding, knockdown of *SGK1* significantly reduced the expression of AP-1 network genes (Fig. 8, *C* and *D*), and depleting the expression of an AP-1 network gene, *EGR1*, abrogated the expression of *NDRG1* (Fig. 9*A*), a downstream component of the pathway, in T47D and MDA-MB-231. Taken together, these results suggest that progesterone and *SGK1* regulate the expression of *NDRG1* via the AP-1 network genes.

### SGK1/NDRG1 axis inactivates the EGFR–mitogen-activated protein kinase pathway to inhibit migration and invasion of breast cancer cells

**Figure 10.**
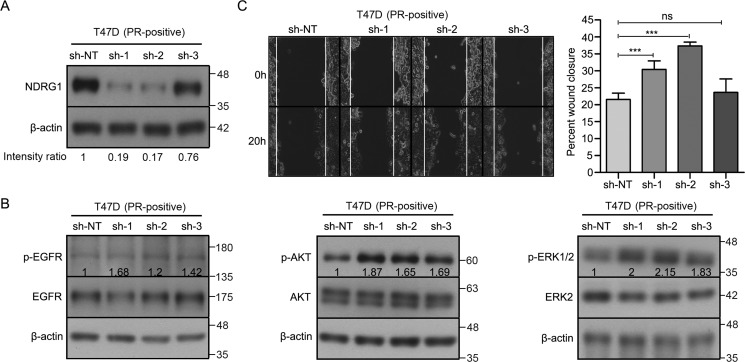
**NDRG1 regulates the activation of multiple cellular kinases and cell migration in T47D cells.** *A*, Western blot analysis depicting knockdown of *NDRG1* in T47D cells (PR-positive). sh-NT was used as vector control for *NDRG1* expression. β-Actin protein was used as a loading control for Western blotting. *Numbers* on the blot indicate intensity ratio for NDRG1 expression normalized to respective β-actin levels. The analysis is representative of three independent experiments. *B*, Western blot analysis of p-EGFR (Tyr-1086), p-AKT (Ser-473), and p-ERK1/2 (Thr-202/Tyr-204) in *NDRG1* knockdown clones of T47D cells. β-Actin used as a loading control for Western blotting. The figure is representative of three independent experiments. *Numbers* on the blot indicate average intensity ratio calculated from all of the three replicate experiments for phosphorylation levels of EGFR, AKT, and ERK1/2, normalized to the respective total protein levels (EGFR, AKT, and ERK2). Western blot analysis is representative of three independent experiments. *C*, cell migration analysis upon depletion of *NDRG1* in T47D breast cancer cells. Cells were monitored by a time-lapse wound healing assay for 20 h. Cell migration from the initial to the 20-h time point was plotted as percentage wound closure (average of the three biological replicate experiments), and the comparison was with respect to sh-NT. The figure is representative of three independent experiments performed in triplicates. *p* value was calculated using Student's unpaired *t* test. ***, *p* < 0.0005; *ns*, not significant. *Error bars* indicate S.D.

**Figure 11.**
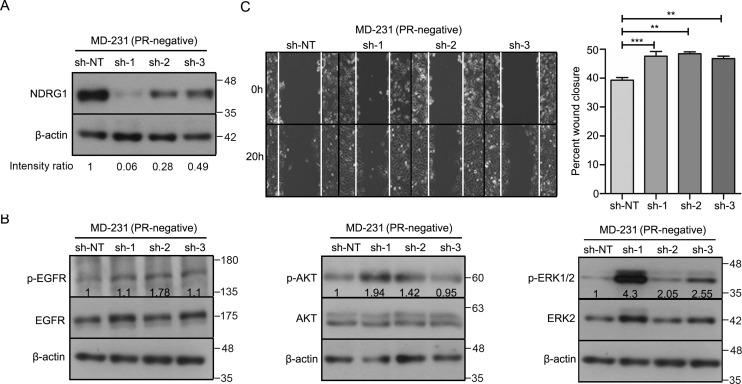
**Depletion of *NDRG1* activates multiple cellular kinases and increases migration of MDA-MB-231 cells.** *A*, Western blot analysis depicting knockdown of *NDRG1* in MD-231 breast cancer cells (PR-negative). sh-NT was used as vector control for *NDRG1* expression. β-Actin protein was used as a loading control for Western blotting. *Numbers* on the blot indicate the intensity ratio for NDRG1, normalized to respective β-actin levels. The analysis is representative of three independent experiments. *B*, Western blot analysis of p-EGFR (Tyr-1086), p-AKT (Ser-473), and p-ERK1/2 (Thr-202/Tyr-204) in *NDRG1* knockdown clones of MD231 cells. β-Actin was used as a loading control for Western blotting, and β-actin for the *p-EGFR* and *p-ERK1/2 panels* is the same. The *numbers* on the blot indicate average intensity ratio calculated from all of the three replicate experiments for phosphorylation levels of EGFR, AKT, and ERK1/2, normalized to respective total protein levels (EGFR, AKT, and ERK2). Western blot analysis is representative of three independent experiments. *C*, migration of cells was measured from 0 to 20 h by using a time-lapse wound healing assay. The bar plot represents percentage wound closure (average of the three biological replicate experiments), and the comparison was with respect to sh-NT. The figure is representative of three independent experiments performed in triplicates. *p* value was calculated using Student's unpaired *t* test. **, *p* < 0.005; ***, *p* < 0.0005; *ns*, not significant. *Error bars* indicate S.D.

We recently showed that progesterone decreases the activation of multiple kinases like EGFR, AKT1, and ERK1/2 in breast cancer cells, leading to suppression of cell migration (19). To test whether *NDRG1* mediates inactivation of EGFR/AKT1/ERK1/2 kinases in response to progesterone, we knocked down the expression of *NDRG1* in T47D and MDA-MB-231 cells (Figs. 10*A* and 11*A*). Interestingly, two of three shRNA clones targeting *NDRG1* significantly increased the phosphorylation of EGFR (Tyr-1068), AKT (Ser-473), and ERK1/2 (Thr-202/Tyr-204) (Figs. 10*B* and 11*B*), which remained unaffected even upon treatment with progesterone, suggesting an essential role of *NDRG1* to mediate progesterone response (Figs. S3 (*A* and *B*) and S4 (*A* and *B*)). Furthermore, breast cancer cells expressing constructs targeting *EGR1* and *NDRG1* displayed an increase in breast cancer cell migration (Figs. 9*B*, 10*C*, and 11*C*). Also, the *NDRG1*-depleted cells continued to show an increased cell migration regardless of progesterone treatment (Figs. S3*C* and S4*C*). Taken together, our results suggest that the *SGK1/NDRG1* axis mediates regulation of activation of kinases involved in breast cancer cell migration, independent of their hormonal receptor status.

## Discussion

**Figure 12.**
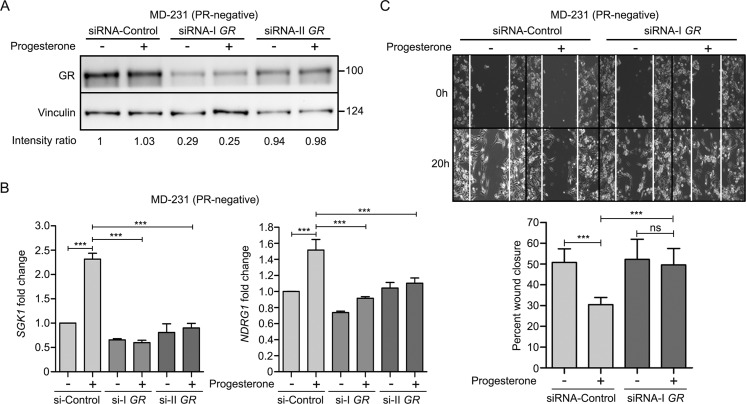
**GR mediates progesterone action in PR-negative breast cancer cells.** *A*, Western blot analysis of GR upon knockdown of *GR* in MDA-MB-231 (PR-negative) cells using two siRNAs targeting *GR*. The cells were treated with (+) and without (−) progesterone. Vinculin was used as an internal protein loading control. The *numbers* on the blot indicate intensity ratio for GR expression normalized to respective vinculin levels. The analysis is representative of three independent experiments. *B*, quantitative real-time PCR for *SGK1* and *NDRG1* in *GR*-depleted MD-231 cells in the presence of progesterone. The bar plot indicates -fold expression of both the genes in each of the siRNAs, compared with respect to expression in siRNA-control. *GAPDH* was used as an internal normalization control. The analysis is representative of three independent experiments performed in triplicates. *C*, cell migration in MD-231 cells expressing siRNA-I targeting *GR* and siRNA-control, treated with (+) and without (−) progesterone, was performed for 21 h. The bar plot indicates percentage cellular migration of the cells upon depletion of *GR* compared with siRNA-control in the presence and absence of progesterone, and the analysis is representative of three independent experiments performed in triplicates. *p* value was calculated using Student's unpaired *t* test. ***, *p* < 0.0005; *ns*, not significant. *Error bars* indicate S.D.

Preoperative endocrine therapies, in contrast to neoadjuvant chemotherapy, are much simpler and more economical to deliver. An understanding of the targets could thus be of immense potential utility in monitoring the response of hormones in human cancer. This study details the underlying molecular mechanism associated with benefits of preoperative progesterone treatment as observed in our randomized trial (13). We present an intricate convergence model indicating a dual-phase regulation downstream to progesterone treatment to regulate the expression of serum- and glucocorticoid-regulated kinase gene 1 (*SGK1*), predominantly driven as a direct transcriptional target, consistent with earlier reports (20, 21), in PR-positive breast cancer cells and down-regulation of *miR-29a* and *miR-101-1* targeting *SGK1* with a relatively distinct effect in PR-negative breast cells in response to progesterone. Furthermore, our analysis suggests that in PR-negative cells, the glucocorticoid receptor (*GR*) mediates the effect of progesterone by regulating the expression of *SGK1* and *NDRG1* and cell migration (Fig. 12). However, the role of membrane progesterone receptors (*PGRMC1* and *SERBP1*), which are uniformly expressed across breast cancer cells (Table 1), remains to be elucidated in these cells. The stringent up-regulation of *SGK1* in response to progesterone led to an activation of a tumor metastasis suppressor gene, *NDRG1*, via a set of AP-1 network genes to inactivate AKT1, ERK1/2, and EGFR kinases, impeding the invasion and migration of breast cancer cells. Moreover, *NDRG1* is known to regulate the activity of *EGFR*, either by decreasing its expression or by impeding its heterodimerization with other ErbB family members (27, 28). Furthermore, NDRG1 also suppresses the activation of downstream targets such as EGFR, AKT, and ERK1/2 kinases (29, 30). Thus, consistent with the literature, we show that *NDRG1* regulates the activation of *EGFR* and downstream kinases in breast cancer in response to progesterone. As *NDRG1* is known to be regulated by AP-1 network genes in response to stress-induced activation of kinases (22–25), this model confirms and extends our recent report that progesterone modulates the effect of surgical stress by up-regulation of *NDRG1* in primary breast cancer patients (26), affecting the invasive characteristics of breast cancer cells most likely by regulating their migration.

**Table 1:** **Selection of breast cell lines and validation of PR/ER/Her2 hormone receptor status** A panel of breast cell lines with varying receptor status, as reported in the literature, was selected for studying the effect of progesterone independent of the receptor status. The PR/ER/Her2 transcript expression of all of the cell lines was confirmed by RT-PCR and using gene expression array analysis. The RT-PCR analysis for PR/ER/Her2 for T47D, MCF7, BT474, ZR-75-1, and MDA-MB-231 was as described earlier (19).

	Breast cell lines	Literature-reported receptor status			Validation of receptor status at expression level								
					Expression array analysis			RT-PCR					
		PR	ER	HER2	PR	ER	HER2	PR	ER	HER2	GR	PGRMC1	SERBP1
1	T47D	+	+	−	+	+	−	+	+	−	+	+	+
2	MCF7	+	+	−	+	+	−	+	+	−	+	+	+
3	BT474	+	+	+	+	+	+	+	+	+	+	+	+
4	ZR-75-1	−	+	−	−	+	−	−	+	−	+	+	+
5	MDA-MB-231	−	−	−	−	−	−	−	−	−	+	+	+
6	184A1 (immortalized cell line)	−	−	−	−	−	−	−	−	−	+	+	+

**Figure 13.**
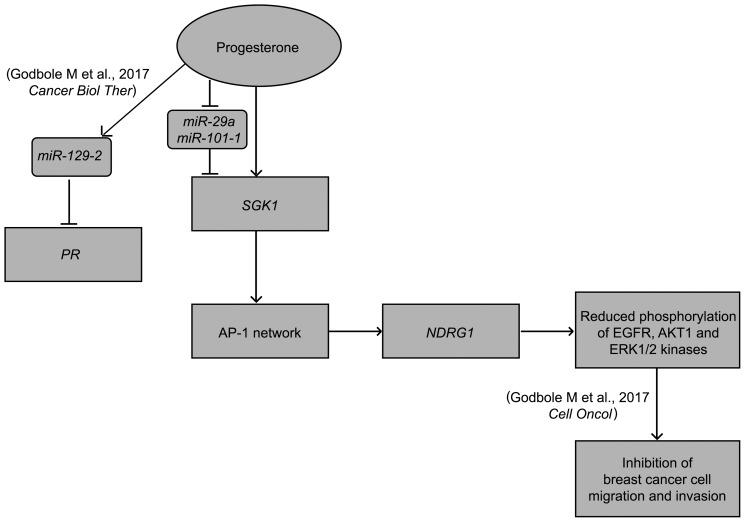
**Model depicting the action of progesterone in breast cancer.** The figure summarizes our study where progesterone treatment of breast cancer cells increases the expression of *SGK1*, which up-regulates *NDRG1* via the AP-1 network genes, independent of the PR status of the cells. We also show that progesterone suppresses the expression of *miR-29a* and *miR-101-1* targeting the 3′-UTR of *SGK1*, a dual-regulatory mode of expression of *SGK1* in breast cancer. The increased expression of *NDRG1* causes reduction in phosphorylation of kinases and thus suppresses cell invasion and cell migration of the cells, thus providing a mechanism to our previous study (19). The model also summarizes our previous results where we have shown that progesterone-mediated up-regulation of *miR-129-2* decreases the expression of *PR* in breast cancer (36). Thus, the model provides a molecular basis to the clinical findings of preoperative progesterone intervention in breast cancer.

Interestingly, *SGK1* and *NDRG1* are known to be down-regulated in human cancers as compared with adjacent normal tissues, and increased expression of both of these genes has been associated with better survival of cancer patients (31–35). Even the recently described panel of 38 gene signatures that predict favorable prognosis of breast cancer patients includes *SGK1* (15). Thus, enhanced expression of *SGK1* and *NDRG1* could explain better survival of breast cancer patients (13). Our study suggests that *SGK1* up-regulates the expression of *NDRG1*, with no significant change in phosphorylation of NDRG1 in breast cancer cells. We describe that *SGK1* regulates the expression of *NDRG1* via regulation of expression of *EGR1*, a transcription factor from the AP-1 network genes, in breast cancer independent of the PR status of cells. In summary, we propose a model for the mode of action of progesterone in breast cancer deciphering the molecular basis of a randomized clinical trial studying the effect of progesterone in breast cancer (Fig. 13). Whereas there have been attempts to understand the effect of progesterone as a physiological hormone (17), our analysis provides mechanistic insights into the role of progesterone in breast cancer, detailing a possible genetic event leading to clinical observation of better survival of breast cancer patients treated with preoperative progesterone (13). However, it remains to be studied whether these molecular targets of progesterone could help in stratification of breast cancer patients and aid in better prognosis.

## Experimental procedures

### Breast cell lines

T47D, BT474, MDA-MB-231, ZR-75-1, and MCF7 breast cancer cell lines and an immortalized normal-like breast cell line 184A1 were obtained as a gift from the laboratory of Dr. Dennis J. Slamon (UCLA, Los Angeles, CA). The cell lines were authenticated by DNA short tandem repeat profiling using the Promega GenePrint 10 system, and analysis was done using the GeneMarker HID software and the ATCC database (Table S3). Cells were tested for mycoplasma and were made mycoplasma-free using EZKill mycoplasma removal reagent (HiMedia). T47D, BT474, MDA-MB-231, MCF7, and ZR-75-1 cells were cultured as described previously (19, 36). The immortalized normal-like 184A1 cell line was cultured in DMEM/F-12 medium (HiMedia) supplemented with 28.18 IU of insulin, 20 ng/ml EGF, and 500 ng/ml hydrocortisone. Basal medium was supplemented with 10% (v/v) fetal bovine serum (Gibco), 2.5 mg/ml Amphotericin-B (Abbott), and 1.25 μl/ml gentamycin (Abbott). All of the cells were cultured at 37 °C in a 5% CO_2_ incubator. The ER/PR/Her2 receptor status of all of the cells was validated by RT-PCR and expression array analysis (Table 1).

### Progesterone treatment and RNA isolation

Breast cancer cells were treated with progesterone, and RNA isolation was performed as described earlier (19, 36). Additionally, in the case of mifepristone + progesterone (M+P) combination treatment, 100 nm RU486 (mifepristone) was added to the cells for 2 h, followed by 10 nm progesterone treatment for 6 h in the same medium. An equal amount of alcohol was used as vehicle control. The treatment conditions for progesterone and mifepristone were standardized based on the expression change of three known candidate genes (Fig. S1*A*).

### Gene expression profiling

Gene expression profiling was performed using the BeadChip Illumina microarray platform. Raw data (.idat files) of the BeadChip Illumina platform were converted to readable format using Genome studio software (version V2011.1). Probe level data were converted into gene-centric estimates and used for further processing. The Bioconductor lumi package was used for preprocessing the data, which includes quality control steps, background correction, normalization, log transformation, etc. and finally differential gene analysis. Robust spline normalization and variance stabilization transformation methods were used for normalization and transformation, respectively. To specifically select for highly variable genes, unexpressed, nonannotated, and false positive genes were excluded from the analysis. Median absolute deviation was carried out to make samples comparable with each other. The differential gene expression cut-off was set as 0.5 ≤ log (-fold change) ≤ −0.5 in at least one of the three conditions (control, progesterone, and mifepristone + progesterone). Heat maps were constructed using MeV software (version 4.9.0) (Fig. S5). All possible comparisons were taken into consideration as progesterone *versus* control (P *versus* C), mifepristone + progesterone *versus* control (M+P *versus* C), and progesterone *versus* mifepristone + progesterone (P *versus* M+P). From our analysis, we identified 623, 553, 1873, 532, 1764, and 4703 differentially expressed genes in T47D, MCF7, BT474, ZR-75-1, MDA-MB-231, and 184A1 breast cell lines, respectively. The microarray raw data are available on ArrayExpress (https://www.ebi.ac.uk/arrayexpress/experiments/E-MTAB-6742/)^5^ (47).

### ChIP-Seq analysis

ChIP-Seq data for PR and p300 pulldown in PR-positive breast cancer cell lines (MCF7 and T47D) was obtained from the gene expression omnibus (37) (GSE68359). The raw data were analyzed to identify differential binding sites upon progesterone treatment. Reads were aligned against the GENCODE human reference genome (GRCh38, release 28) (38) using BWA (version 0.7.17) (39). Reads with an alignment quality score less than 15 were filtered using SAMtools (40). MACS2 (41) was used for peak calling from individual replicates to identify PR-binding sites. Further processing of the peaks was performed using DiffBind (42), a bioconductor package to determine replicate clustering, formulation of consensus peak sets, and identification of differential binding sites. A false discovery rate cut-off of 0.0001 was used to identify reliable sites. Annotation was performed for the ±5-kb window of the PR- and p300-binding genomic regions using the UROPA tool (43).

### RNA-Seq analysis

Analysis of RNA-Seq–based gene raw counts for T47D and MCF7 cell lines, untreated and treated with progesterone (obtained from GSE68358), was performed using R language. DESeq2 bioconductor package (44) was used to identify the differentially expressed genes.

### Integrated analysis

A weighted gene co-expression network was constructed based on the gene expression data to identify gene modules up-regulated in response to progesterone (45) across all of the breast cancer cells. *SGK1* and *NDRG1* were found to be the top up-regulated and recurrent gene in response to progesterone across multiple cells (Fig. S6). Next, we analyzed our small RNA-Seq data to identify differentially expressed microRNA in response to progesterone treatment predicted to bind to the 3′-UTR of the *SGK1* gene using six different microRNA binding site prediction tools (36). Bioinformatics prediction analysis revealed identification of *miR-29a* and *miR-101-1* to target the 3′-UTR of *SGK1* that was down-regulated in response to progesterone treatment.

### Small RNA-Seq analysis

To identify the microRNA's targeting *SGK1*, we analyzed our small RNA-Seq data, described earlier (36). The sequencing was performed on a single lane of the Illumina HiSeq 1000 platform with four breast cancer cell lines (T47D, BT474, MCF7, and MDA-MB-231). For identifying microRNAs targeting 3′-UTR of *SGK1*, differentially expressed microRNAs in response to progesterone were overlapped with microRNAs predicted to target *SGK1* using the six algorithms used in our earlier study (36).

### Quantitative real-time PCR

Transcript levels of candidate genes and microRNAs were analyzed by quantitative real-time PCR as described previously for genes and microRNAs (19, 36). Briefly, cDNAs from each cell line with the three conditions (control, progesterone, and M+P) were then subjected to qRT-PCR analysis. The *GAPDH* gene was used as an internal control, and an average of *C_t_* values from each condition was used to normalize the *C_t_* values of candidate genes in each cell line. In the case of microRNAs, *U6* small RNA was used as an internal control for normalizing the expression of microRNAs. Expression change of candidate genes and microRNAs was calculated by the 2^−ΔΔ*Ct*^ method. Primer sequences used for real-time PCR validation of genes and microRNAs are provided in Table S4. Sequences for real-time PCR primers against *PR* have been provided earlier (36).

### Overexpression and knockdown studies

For overexpression of *SGK1*, a retrovirus-based pBABE-puro-*SGK1* construct that expresses WT *SGK1* (WT-*SGK1*) was used. Untransfected parent cells were used as control for overexpression. Positive clones were selected using 1 μg of puromycin. Overexpression experiments were performed in T47D (PR-positive) and MDA-MB-231 (PR-negative) breast cancer cells. For the knockdown of *SGK1*, *NDRG1*, and *EGR1* genes in T47D and MDA-MB-231 cells, three lentiviral shRNA constructs (PLATINUM Select Human MLP lentiviral shRNA-mir vector, Transomic Technologies) each, against these genes, were used for genetic depletion. Positive clones were selected using 1 μg of puromycin. For transient knockdown of the progesterone receptor (*PR* or *NR3C3*), three lentiviral shRNAs targeting the *PR* were used. 48 h post-transduction, T47D cells, without selection, were used for further experimentation. The short hairpin-nontargeting (sh-NT) was used as vector/scrambled control for all knockdown experiments.

### Knockdown of GR

A transient siRNA-mediated knockdown of the *GR* (*NR3C1*) was performed in PR-negative MDA-MB-231 cells. Two siRNAs targeting *GR* were used, along with one siRNA-control (all siRNAs were from Cell Signaling Technology) to compare the expression of *GR* and downstream targets. Following siRNA transfection for 72 h using Lipofectamine RNAiMAX (Thermo Fisher Scientific), cells were treated with progesterone as detailed above and processed for RNA isolation and protein sample preparation.

### Protein sample preparation and Western blot analysis

Protein samples were prepared, and Western blots were developed as described earlier (19). Briefly, cells were serum-starved and treated with progesterone for 8 h or left untreated. Cell lysates were prepared, and equal amounts of lysate were resolved using 10% SDS-PAGE and transferred to a polyvinylidene difluoride membrane by the wet-transfer method. The immunoblots were then incubated with primary antibodies against SGK1 (Cell Signaling Technology, 1210S; dilution 1:800); p-SGK1 (Abcam, ab55281; dilution 1:500); NDRG1 (Cell Signaling Technology, 9485S; dilution 1:800); p-NDRG1 (Cell Signaling Technology, 5482S; dilution 1:800); EGR1 (Santa Cruz Biotechnology, Inc., sc-515830; dilution 1:1000); β-actin (I-19)-R (Santa Cruz Biotechnology, sc-1616-R; dilution 1:3000); p-EGFR (Tyr-1068) (Cell Signaling, 3777S; dilution 1:500), p-Akt (Ser-473) (Cell Signaling, 4060S; dilution 1:500); p-ERK1/2 (Thr-202/Tyr-204) (Cell Signaling, 9101S; dilution 1:1000); EGFR (1005) (Santa Cruz Biotechnology, sc-03; dilution 1:1000); AKT (11E7) (Cell Signaling, 4685S; dilution 1:1000); ERK2 (c-14) (Santa Cruz Biotechnology, sc-154; dilution 1:1000); GR (Cell Signaling Technology, 3660S; dilution 1:1000); and vinculin (Cell Signaling Technology, 4650S; dilution 1:4000). Goat anti-rabbit IgG-horseradish peroxidase (Santa Cruz Biotechnology, sc-2004; dilution 1:3000) and goat anti-mouse IgG-horseradish peroxidase (Santa Cruz Biotechnology, sc-2005; dilution 1:3000) were used as secondary antibodies.

### Treatment with SGK1 inhibitor

Cells were grown until 70–80% confluence in a 6-well dish and then serum-starved using low-glucose DMEM (HiMedia) for 24 h. 1.0 μm concentration of GSK650394A (SGK1 inhibitor, Tocris) was added to the respective wells for 4 h, as discussed in other studies (46). After 4 h, medium was removed and fresh low-glucose medium was added to cells. Where indicated, cells were then treated with 10 nm progesterone for 6 h and then used for RNA or protein isolation.

### Cell invasion assay

Cell invasion assay was performed as described earlier (19). Briefly, 35,000 cells were allowed to invade Matrigel in Boyden chambers (Corning) for 16–18 h at 37 °C. Cells were observed under an upright microscope, 10 random fields were chosen, and the number of cells in each field were counted and plotted as percentage cell invasion.

### Wound-healing assay

Wound scratch migration assay and analysis was performed as described earlier (19). Briefly, confluent cell monolayer in a 6-well plate was subjected to a scratch manually with a sterile small pipette tip. Cell culture medium was replaced with low-glucose phenol-red free DMEM containing 10% charcoal-stripped fetal bovine serum. Cell migration was monitored for 20 h, and distance traversed by cells was quantified.

### Dual-Luciferase assay with microRNAs/SGK1 3′-UTR

Cloning of microRNA sequences and *SGK1* 3′-UTR was performed as described earlier (36). Briefly, a 400-bp sequence of *miR-29a* and *miR-101-1* containing the seed sequence was PCR-amplified using genomic DNA isolated from T47D. Amplicons were cloned in a T/A cloning vector (Fermentas), followed by subcloning in BamHI and HindIII sites of pCDNA 3.1(−) expression vector (Invitrogen). *SGK1*-3′-UTR of 1000 bp was PCR-amplified using T47D cDNA. Amplicons were cloned in a T/A cloning vector followed by subcloning between XbaI sites in a pGL3-promoter vector (Luciferase Expressing vector, Promega). For the Dual-Luciferase assay, 293FT cells (50,000 cells/well) were transfected using Lipofectamine 2000 reagent (Thermo Fisher Scientific) with a combination of these constructs along with a *Renilla* luciferase vector (for normalizing transfection efficiency) in separate wells. 5 nm miR inhibitors (anti-miRs) (Sigma) were also transfected in combination to expression vectors for specifically inhibiting the activity of both of the microRNAs. 48 h post-transfection, cells were lysed, and a luciferase assay was performed to measure firefly luciferase activity after normalization to *Renilla* luciferase values (Centro LB 960, Multimode Microplate Reader, Berthold Technologies, Bad Wildbad, Germany). The experiment was performed in triplicates, and differences between groups showing *p* values of <0.05 (calculated using an unpaired Student's *t* test) were considered significant.

### Transfection of microRNA inhibitor in breast cancer cells

T47D cells were grown up to 60% confluence and transfected with 25 nm negative control miR inhibitor (anti-*miR-129-2*), anti-*miR-29a*, and anti-*miR-101-1* (Sigma). Post-transfection, cells were incubated for 48 h and then treated with progesterone for 6 h as described above. Cell lysate was prepared, and Western blot analysis was performed to study expression of SGK1.

### Statistical analysis

Statistical analysis was performed using GraphPad Prism version 5 software (GraphPad Software, Inc., La Jolla, CA). Student's unpaired *t* test was used to determine the statistical significance.

## Author contributions

M. G. and A. D. designed the experiments. M. G., T. T., K. P., B. D., N. Y., S. J., N. G., K. T., P. T., S. D., R. P., H. D., S. S., K. K., D. K., and P. C. performed the experiments. S. D., S. G. and R. A. B. provided the reagents. M. G. and A. D. wrote the paper.

## Supplementary Material

Supporting Information

## Footnotes

5 Please note that the JBC is not responsible for the long-term archiving and maintenance of this site or any other third party hosted site.

## Footnotes

4 The abbreviations used are:

PR: progesterone receptor
ER: estrogen receptor
EGFR: epidermal growth factor receptor
GR: glucocorticoid receptor
DMEM: Dulbecco's modified Eagle's medium
M+P: mifepristone + progesterone
sh-NT: short hairpin-nontargeting
p-: phosphorylated
miR: microRNA.
