# Supplementary material for: Up-regulation of the kinase gene SGK1 by progesterone activates the AP-1–NDRG1 axis in both PR-positive and -negative breast cancer cells
Source: J Biol Chem. 2018 Oct 18;293(50):19263–76. doi: 10.1074/jbc.RA118.002894 (PMC6298595; doi:10.1074/jbc.RA118.002894)
Supplement: Supporting Information [file supp_293_50_19263__index.html]

Up-regulation of the kinase gene SGK1 by progesterone activates the AP-1–NDRG1 axis in both PR-positive and -negative breast cancer cells — SGK1/AP1/NDRG1 mediates progesterone effect in breast cancer — Up-regulation of the kinase gene SGK1 by progesterone activates the AP-1–NDRG1 axis in both PR-positive and -negative breast cancer cells — SGK1/AP1/NDRG1 mediates progesterone effect in breast cancer — Supporting Information 

# Up-regulation of the kinase gene *SGK1* by progesterone activates the AP-1–NDRG1 axis in both PR-positive and -negative breast cancer cells

## Supporting Information

- Godbole et al\_Supplementary information - Supplementary tables and figures
