# Supplementary material for: Up-regulation of the kinase gene SGK1 by progesterone activates the AP-1–NDRG1 axis in both PR-positive and -negative breast cancer cells
Source: J Biol Chem. 2018 Oct 18;293(50):19263–76. doi: 10.1074/jbc.RA118.002894 (PMC6298595; doi:10.1074/jbc.RA118.002894)
Supplement: Supporting Information [file supp_RA118.002894_136628_3_supp_221065_pgsmw6.pdf]

Supplementary information:

Table S1: Relative log-fold change of expression of genes-of-interest in response to progesterone as identified in microarray gene expression analysis and fold change from real-time PCR analysis

	Gene name	T47D	BT474	MCF7	ZR-75-1	MDA-MB-231
**Microarray**	*SGK1*	3.510	2.818	0.3748	0.6782	-0.0945
	NDRG1	1.818	1.14	0.0890	0.5606	0.2172
**Real-time PCR**	*SGK1*	22.9801	10.0952	1.7566	4.7698	2.1947
	NDRG1	3.4690	2.1108	4.5726	3.5433	1.8818

Table S2: Relative log-fold change of expression of microRNAs-of-interest in response to progesterone as identified in small RNA sequencing and fold change from real-time PCR analysis

	MicroRNA name	T47D	BT474	MDA-MB-231
**Small RNA sequencing**	*miR-29a*	-1.605	-1.713	N.D.
	miR-101-1	-1.605	-1.713	N.D.
**Real-time PCR**	*miR-29a*	0.9001	1.1217	0.8274
	miR-101-1	0.8074	1.2204	0.4402

N.D: not determined in small RNA sequencing

Table S3: STR profiling of breast cell lines

Query for STR	10-marker STR profile as per GeneMarker HID software											Authenticity of cell line as identified
	**TH01**	**D21S11**	**D5S818**	**D13S317**	**D7S820**	**D16S539**	**CSF1PO**	**AMEL**	**vWA**	**TPOX**	**% Match**	
T47D	6	28,31	12	12	11	10	11,13	X	14	11	100	T47D
BT-474	7,8	28,32.2	11,13	11	9,12	9,11	10,11	X	15,16	8	100	BT-474
MCF7	6	30	11,12	11	8,9	11,12	10	X	14,15	9,12	100	MCF7
MD-231	7,9.3	30,33.2	12	13	8,9	12	12,13	X	15,18	8,9	100	MD-231
ZR-75-1	8,9.3	29	13	9	8,11	11	10,11	X	16,18	8	92%	ZR-75-1
184A1	9.3	29,30	11,13	11	9,11	11,12	10,11	X	18,19	11	100	184A1

Table S4: Primer sequences used for real-time PCR validation of genes and microRNAs

Sr. No.	OAD Number	Primer sequence	Gene/ microRNA name
1.	OAD 137	5’ GGCGCTTCAGGCACTACAA	*F3*
	OAD 138	3’ TTGATTGACGGGTTTGGGTTC	
2.	OAD 139	5’ GCAGAGTCCGTGACAGAGG	*STAT5A*
	OAD 140	3’ CCACAGGTAGGGACAGAGTCT	
3.	OAD 141	5’ CCCACATGAAGCGACTTCCC	*EZF*
	OAD 142	3’ CAGGTCCAGGAGATCGTTGAA	
4.	OAD 233	5’ GCAGAAGAAGTGTTCTATGCAGT	*SGK1*
	OAD 234	3’ CCGCTCCGACATAATATGCTT	
5.	OAD 457	5’ GCCTCCTTCCCCGCAGGG	*NDRG1*
	OAD 458	3’ GCCCAAACTGTTGAAGGACTCC	
6.	OAD 567	5’ GCATTGGCAGGAGGGGCAAGG	*FOS*
	OAD 568	3’ CAGCTCCCTCCTCCGGTTGCG	
7.	OAD 577	5’ CCCAAGAACGTGACAGATGAG	*JUN*
	OAD 578	3’ TGCCCCGTTGACCGGCTGC	
8.	OAD 573	5’ GGCGAGCAGCCCTACGAGC	*EGR1*
	OAD 574	3’ GTATAGGTGATGGGGGGCAGTC	
9.	OAD 571	5’ CCTGCAGTACCCCACTCTACG	*DUSP1*
	OAD 572	3’ CCCAAGGCATCCAGCATGTCC	
10.	OAD 328	5’ AATCCCATCACCATCTTCCA	*GAPDH*
	OAD 329	3’ GGACTCCACGACGTACTCA	
11.	OAD 552	5’ TAGCACCATCTGAAATCGGTTA	*miR-29a*
12.	OAD 625	5’ CAGTTATCACAGTGCTGATGCT	*miR-101-1*

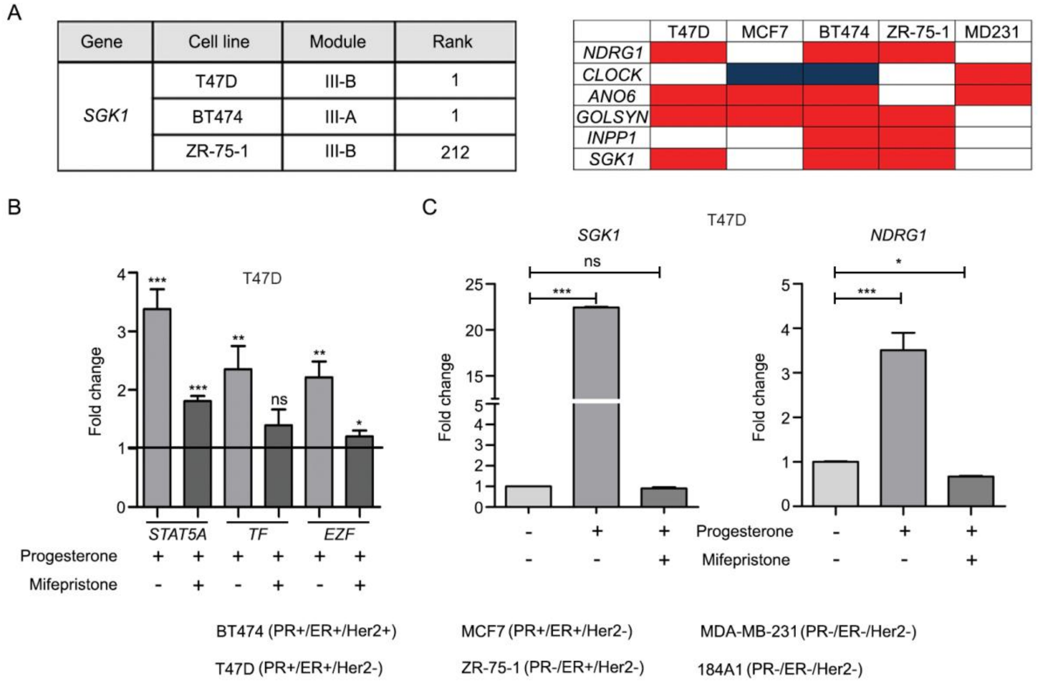

Figure S1: Identification and validation of candidate genes and standardization of progesterone treatment in breast cancer cells

A) Representation of expression array results; left panel indicates the WGCNA results with *SGK1* being the top up-regulated gene in cells indicated, in response to progesterone. Figure panel on the right indicates results of the recurrent gene expression analysis. Genes de-regulated in more than three cell lines were considered to be recurrently expressed in response to progesterone. Red colour in the figure indicates up-regulation, blue colour indicates down-regulation, and no colour/blank stands for no change in gene expression in response to progesterone treatment. **B)** Standardization of progesterone treatment was based on expression changes of three known progesterone regulated genes (*STAT5A*, *EZF* and *F3*), studied using quantitative real time PCR analysis. Data has been plotted as expression change in response to progesterone or mifepristone+progesterone treatment, with respect to control (horizontal black line). Figure is representative of three independent experiments performed in triplicates in T47D cells (PR-positive). **C)** Real time PCR validation for expression of *SGK1* and *NDRG1* in T47D cells (PR-positive) in response to progesterone and mifepristone+progesterone treatment. Data has been plotted as fold change for individual gene with respect to expression in control cells and normalized with respect to expression of *GAPDH*. Figure is representative of three independent experiments performed in triplicates. *P*-value was calculated using student's unpaired t-test. * indicates $P<0.05$; ** indicates $P<0.005$; *** indicates $P<0.0005$; ns indicates not significant.

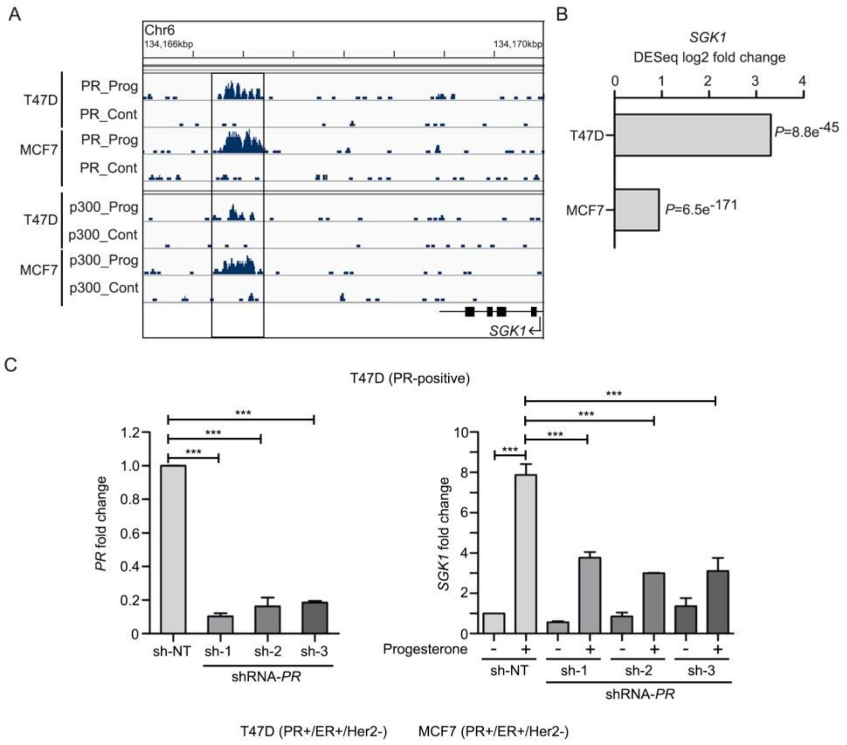

Figure S2: *PR* up-regulates expression of *SGK1* in response to progesterone in PR-positive cells
A) Analysis of the ChIP-Seq data (GSE68359) to identify binding of PR and p300 (active transcription) was performed in T47D and MCF7 (both PR-positive) cells treated with or without progesterone. Figure indicates binding of PR and p300 at genomic locus downstream of *SGK1* gene. The figure corresponds to one representative assay from a total of three independent experiments performed in duplicates. **B)** RNA-Seq analysis was performed to analyze expression change of *SGK1* in T47D and MCF7 cells upon progesterone treatment (GSE68359). The bar plot indicates DESeq log2 fold change for *SGK1* in response to progesterone treatment. The analysis is representative of three independent experiments performed in triplicates. Adjusted *P*-value was obtained by multiple testing using Benjamini-Hochberg method. **C)** Quantitative real time PCR analysis for *PR* and *SGK1* upon knockdown of *PR* in T47D (PR-positive) breast cancer cells. For studying expression of *SGK1*, cells were treated with (indicated by "+") and without (indicated by "-") progesterone. Bar plots indicate fold expression of both the genes, in each of the shRNA-*PR* clone, compared to expression in the sh-NT clone. *GAPDH* was used as an internal normalization control. The analysis is representative of three independent experiments performed in triplicates. *P*-value was calculated using student's unpaired t-test. *** indicates *P* <0.0005.

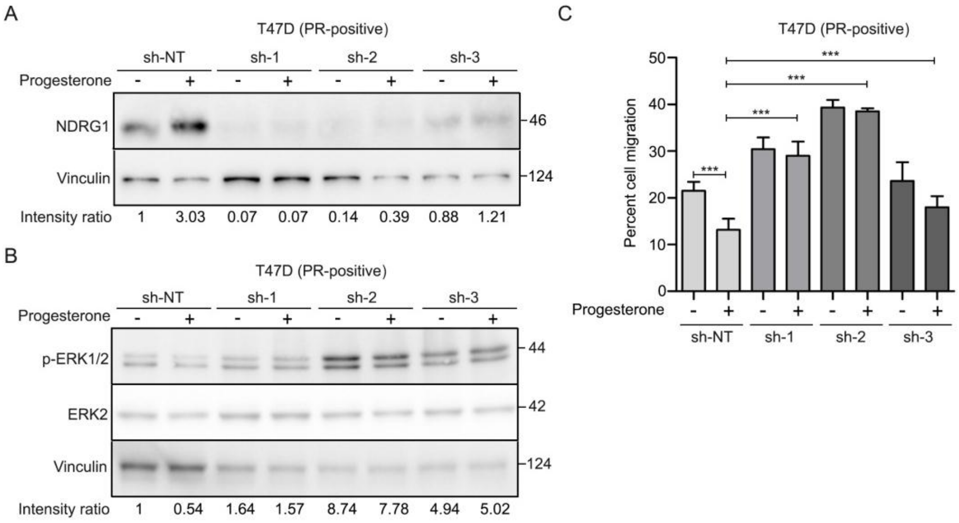

Figure S3: Knockdown of *NDRG1* hampers the effect of progesterone on activation of ERK1/2 and cell migration in T47D cells

A) Western blot analysis depicting the expression of NDRG1 in the *NDRG1*-depleted T47D PR-positive cells treated with (indicated by "+") and without (indicated by "-") progesterone. sh-NT was used as vector control for NDRG1 expression. Vinculin protein was used as loading control for the western blot. Numbers on the blot indicate intensity ratio for NDRG1 expression normalized to respective vinculin levels. The analysis is representative of three independent experiments. **B)** Western blot analysis of p-ERK1/2 (T202/Y204) in *NDRG1* knockdown clones of T47D cells treated with or without progesterone. Vinculin was used as loading control for western blot. Figure is representative of three independent experiments. Numbers on the blot indicate average of intensity ratios calculated from all the three replicate experiments for p-ERK1/2 normalized to ERK2. Western blot analysis is representative of three independent experiments. **C)** Cell migration analysis upon depletion of *NDRG1* in T47D breast cancer cells treated with and without progesterone. Cell migration from initial to 20h time-point was plotted as percentage cell migration, and the bar plot is an average quantification from the three replicates compared with cell migration in sh-NT clone. Figure is representative of three independent experiments performed in triplicates. *P*-value was calculated using student's unpaired t-test. *** indicates $P<0.0005$.

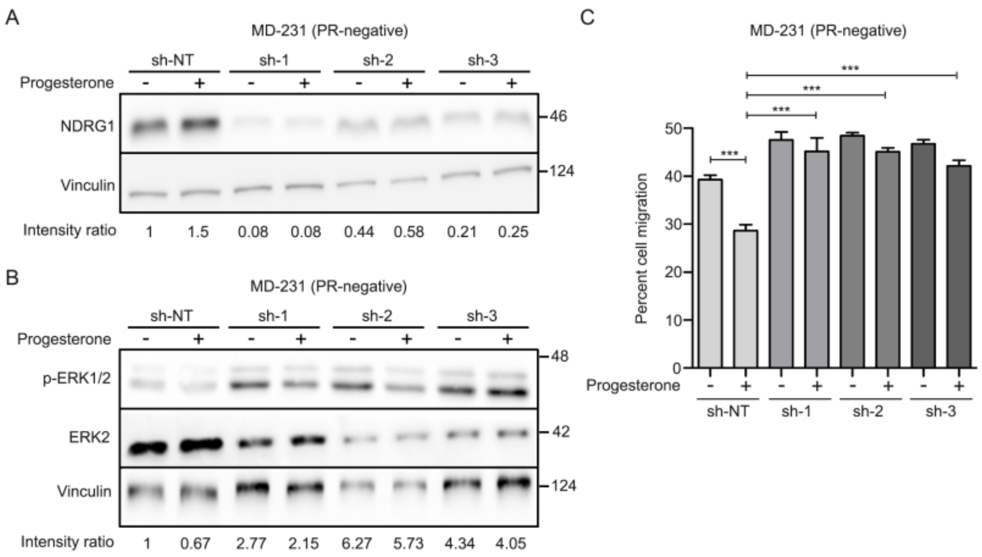

Figure S4: Knockdown of *NDRG1* hampers the effect of progesterone on the activation of ERK1/2 and cell migration in MDA-MB-231 PR-negative cells

A) Western blot analysis depicting the expression of NDRG1 in the *NDRG1*-depleted MD-231 cells treated with (indicated by "+") and without (indicated by "-") progesterone. sh-NT was used as vector control for NDRG1 expression. Vinculin protein was used as loading control for the western blot. Numbers on the blot indicate intensity ratio for NDRG1 expression normalized to respective vinculin levels. The analysis is representative of three independent experiments. **B)** Western blot analysis depicting p-ERK1/2 (T202/Y204) levels in *NDRG1* knockdown clones of MD-231 cells treated with or without progesterone. Vinculin was used as loading control for western blot. Figure is representative of three independent experiments. Numbers on the blot indicate average of intensity ratios calculated from all the three replicate experiments for p-ERK1/2 normalized to ERK2. Western blot analysis is representative of three independent experiments. **C)** Cell migration analysis upon depletion of *NDRG1* in MD-231 PR-negative cells treated with and without progesterone. Cell migration from initial to 20h time-point was plotted as percentage cell migration, and the bar plot is an average quantification from the three replicates compared with cell migration in sh-NT clone. Figure is representative of three independent experiments performed in triplicates. *P*-value was calculated using student's unpaired t-test. *** indicates *P*<0.0005.

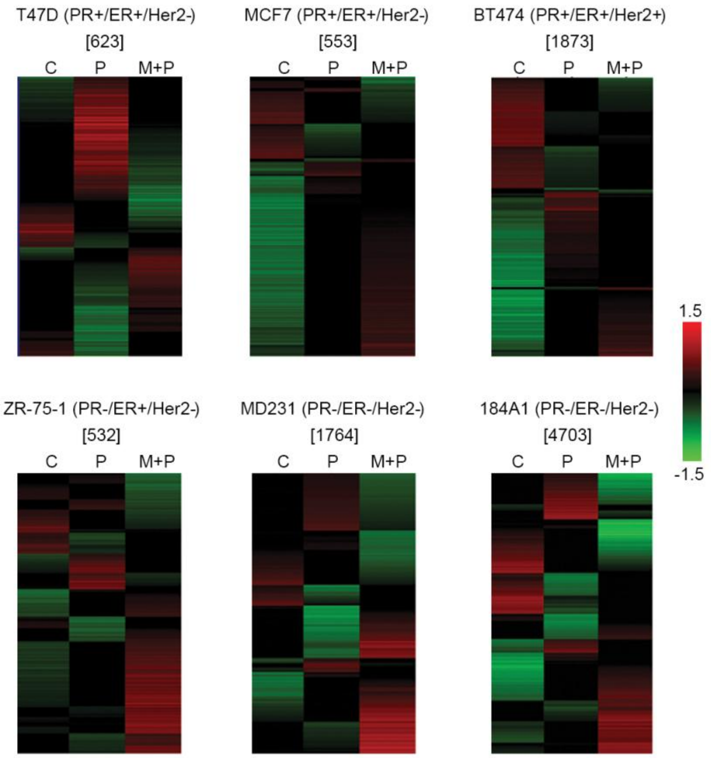

Figure S5: Gene expression profile of breast cell lines with different receptor statuses
Heat map representation of gene expression profile for each breast cell line (n=6) has been shown, with three treatment conditions *viz*. control, progesterone or mifepristone+progesterone (M+P). In the figure, red lines indicate up-regulation; black lines indicate no change; green lines indicate down-regulation of gene in response to the treatment conditions. The differential gene expression cut-off was set as $1.5 \leq \log$ (Fold Change) ≤ -1.5. Numbers in square bracket indicate total number of differentially expressed genes for each cell line.

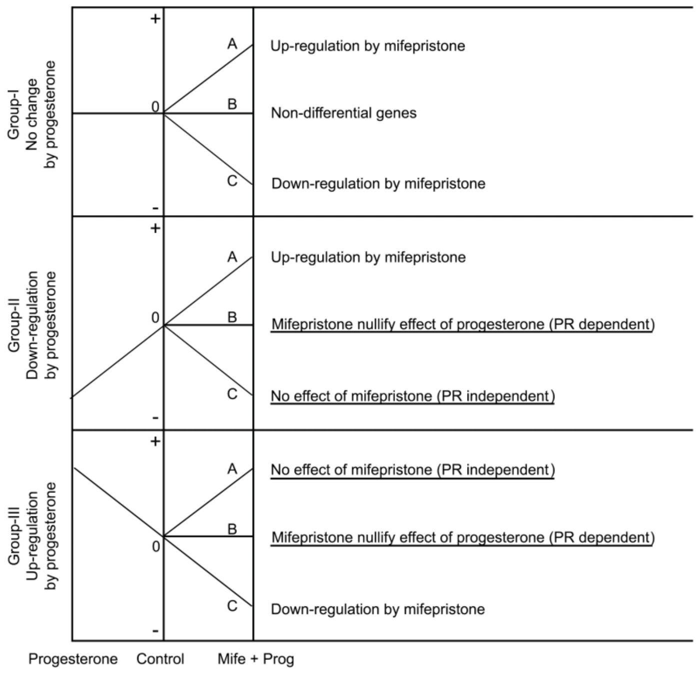

Figure S6: Hypothetical model for all possible pattern of gene expression in microarray analysis with three treatment conditions

To study the pattern of gene expression, a hypothetical model was constructed. Comparison of expression changes in progesterone treated or the combined mifepristone+progesterone (Mife+Prog) treatment are to be compared with the central line of control. Depending on the up-regulation or down-regulation of any gene, the possible outcomes upon the Mife+Prog treatment have been shown in Groups- I, II and III. The subgroups of biological interest are highlighted with an underline.
